# Impact of Morphotype on Image Quality and Diagnostic Performance of Ultra-Low-Dose Chest CT

**DOI:** 10.3390/jcm10153284

**Published:** 2021-07-26

**Authors:** Anne-Claire Ortlieb, Aissam Labani, François Severac, Mi-Young Jeung, Catherine Roy, Mickaël Ohana

**Affiliations:** 1 Radiology Department, Institut Paoli-Calmettes, 232 Boulevard de Sainte-Marguerite, 13009 Marseille, France; anneclaireor@gmail.com; 2 Radiology Department, Nouvel Hôpital Civil, 1 Place de l’Hôpital, 67000 Strasbourg, France; aissam.labani@chru-strasbourg.fr (A.L.); Mi-Young.Jeung@chru-strasbourg.fr (M.-Y.J.); catherine.roy@chru-strasbourg.fr (C.R.); 3 Biostatistics Department, Nouvel Hôpital Civil, 1 Place de l’Hôpital, 67000 Strasbourg, France; francois.severac@chru-strasbourg.fr; 4 ICube Laboratory, 300 Boulevard Sébastien Brandt, 67400 Illkirch Graffenstaden, France

**Keywords:** multidetector computed tomography, radiation dosage, lung, helical computed tomography

## Abstract

Objectives: The image quality of an Ultra-Low-Dose (ULD) chest CT depends on the patient’s morphotype. We hypothesize that there is a threshold beyond which the diagnostic performance of a ULD chest CT is too degraded. This work assesses the influence of morphotype (Body Mass Index BMI, Maximum Transverse Chest Diameter MTCD and gender) on image quality and the diagnostic performance of a ULD chest CT. Methods: A total of 170 patients from three prior prospective monocentric studies were retrospectively included. Renewal of consent was waived by our IRB. All the patients underwent two consecutive unenhanced chest CT acquisitions with a full dose (120 kV, automated tube current modulation) and a ULD (135 kV, fixed tube current at 10 mA). Image noise, subjective image quality and diagnostic performance for nine predefined lung parenchyma lesions were assessed by two independent readers, and correlations with the patient’s morphotype were sought. Results: The mean BMI was 26.6 ± 5.3; 20.6% of patients had a BMI > 30. There was a statistically significant negative correlation of the BMI with the image quality (ρ = −0.32; IC95% = (−0.468; −0.18)). The per-patient diagnostic performance of ULD was sensitivity, 77%; specificity, 99%; PPV, 94% and NPV, 65%. There was no statistically significant influence of the BMI, the MTCD nor the gender on the per-patient and per-lesion diagnostic performance of a ULD chest CT, apart from a significant negative correlation for the detection of emphysema. Conclusions: Despite a negative correlation between the BMI and the image quality of a ULD chest CT, we did not find a correlation between the BMI and the diagnostic performance of the examination, suggesting a possible use of the ULD protocol in obese patients.

## 1. Introduction

Computed Tomography (CT) accounts for the large majority of medical exposures to ionizing radiation [1]. In chest imaging, the radiation dose delivered varies significantly between clinical indications, machine and institution [2] and the dose-length product (DLP) is estimated at 347 mGy·cm (50th percentile) in a 2017 review of 159,909 US patients [3]. Significant susceptible organs such as the breasts, lungs and thyroid are included in the field-of-view; this justifies the reduction in the radiation dose delivered to the minimum needed for adequate diagnosis [4,5].

Chest CT is an appropriate candidate to a significant radiation dose reduction, due to an overall low attenuation of the lung parenchyma resulting in a high contrast. Substantial efforts have been made to optimize the dose delivered in chest imaging, particularly through the implementation of iterative reconstruction [6] and deep learning reconstruction techniques [7]. It is now possible to achieve a diagnostic chest CT at the radiation dose of a posteroanterior and lateral chest radiograph series [8,9], and this has been proven efficient for various clinical scenarios [10], such as lung nodule detection and follow-up [11,12], pulmonary infection [13], CT-guided percutaneous biopsy [14], lymphangioleiomyomatosis [15], etc. However, the image quality of these Ultra-Low-Dose (ULD) chest CTs remains dependent on the patient’s morphotype, and prior studies [11,16] seldomly included patients with a Body Mass Index (BMI) greater than 30 kg·m^−2^. Consequently, the diagnostic performance of a ULD chest CT is not well documented in obese patients [17].

We hypothesize that beyond a certain morphotype, defined by a threshold of BMI and/or a threshold of Maximum Transverse Chest Diameter (MTCD) and/or gender (given the different distribution of fat and breasts between women and men), the deterioration of the image quality becomes too important and leads to a decrease in the diagnostic performance.

The objective of this study was, therefore, to determine the influence of these morphological parameters (BMI, MTCD and gender) on the objective and subjective image quality of a ULD chest CT and on its diagnostic performance for the detection of nine predefined pulmonary parenchymal lesions.

## 2. Materials and Methods

### 2.1. Population

All 170 patients from 3 prior prospective studies performed in Strasbourg University Hospital and carried out on the same second-generation 320-row scanner (Aquillion One Vision Edition, Toshiba, Japan), were retrospectively included. The first study [18] included 55 patients from July 2013 to May 2014 with an occupational exposure to asbestos of at least 15 years, referred for screening of asbestos-related pleuro-pulmonary lesions. The second study [19] (April 2014 to September 2014) and the third study [20] (April 2015 to September 2015) included 51 and 64 patients, respectively, that were referred for a clinically indicated unenhanced chest CT.

The inclusion criterion common to the 3 studies was an age greater or equal to 35 years for men and 40 years for women. The common exclusion criteria were pregnancy, the inability to maintain apnea for more than 5 s, the inability to raise the arms above the head and the inability to give informed consent.

The renewal of consent was not required for this retrospective study by our hospital institutional review board. Written informed consent and approval from the local Ethics Committee had been obtained in the first instance for all patients, at the time of their initial prospective inclusion in one of the 3 trials.

Age, gender, weight, height, BMI and clinical indication for chest CT were recorded at the time of the examination. The Maximum Transverse Chest Diameter (MTCD) was measured by a radiologist (AC, with 4 years of experience in chest CT) on a mediastinal window, from lateral thoracic wall to lateral thoracic wall.

### 2.2. CT Acquisition

Each patient underwent an unenhanced chest CT with two successive acquisitions: one “full dose” (FD) acquisition (120 kV, automated tube current modulation maxed at 700 mA) used as the Gold Standard and one “Ultra-Low-Dose” (ULD) acquisition (135 kV, 10 mA fixed) [21]. Acquisitions were made on a second-generation 320-row scanner (Aquillion One Vision Edition, Canon), with the parameters given in Table 1. All examinations were performed in successive end-inspiratory apneas, arms raised above the head. The 55 patients from the first study were acquired in prone position, to avoid gravity-dependent posterior parenchymal abnormalities—as recommended when screening for asbestos-related diseases [18]. The 115 patients from the two following studies were acquired in supine position. The posterior–anterior and lateral topograms were used to define the acquisition field, extending from the pulmonary apexes to the diaphragm; the acquisition length was kept identical for the two consecutive FD and ULD acquisitions.

The FD and ULD acquisitions were reconstructed in lung window (width = 1500 HU; center = −700 HU) with a hard kernel and a slice thickness of 1 mm, using the constructor’s iterative reconstruction algorithm set at a standard level (Adaptative Iterative Dose Reduction using 3 Dimensional AIDR-3D, Canon).

The whole set of 340 parenchymal reconstructions were anonymized and randomized into two series of 170 FD and 170 ULD examinations, in a different random order for both.

### 2.3. Dosimetry

Radiation doses were expressed in dose-length product (DLP). The effective radiation dose (ED) was calculated by multiplying the DLP by the chest-specific conversion factor of 0.014 mSv/mGy·cm [22].

### 2.4. Quantitative Image Quality

Image noise was defined as the standard deviation (SD) of the attenuation of air within the tracheal lumen, using a circular region of interest (ROI) averaging at least 40 mm^2^, 1 cm above the carina, in the parenchymal reconstruction. It was measured three times by the same reader (AC), with size and position of the ROI kept constant between patients. The average of the three measurements was used as the final value in the statistical analysis.

### 2.5. Qualitative Image Quality

The overall subjective image quality of ULD acquisitions was assessed independently by two radiologists that specialized in chest imaging (M.O., with 9 years of experience in chest CT and A.C., with 4 years of experience) using a 5-point Likert scale (Table 2), after a training session with joint evaluation of 15 cases.

### 2.6. Diagnostic Performance

Each radiologist independently read the ULD then the FD lung parenchyma acquisitions, both in a different random order and with a delay of at least two weeks in between to limit the risk of a memorization bias.

All images were analyzed on a dedicated Workstation (Vitrea version 6.4, Vital Images), with use of multiplanar reconstructions and Maximum Intensity Projection. For each acquisition, the presence or absence of 9 predefined parenchymal lesions, based on the Fleischner Society lexicon [23] was rated in a binary fashion (i.e., presence or absence of the anomaly). The following lesions were assessed: solid nodule ≥ 5 mm, ground glass nodule, mass ≥ 3 cm, ground glass opacity, alveolar consolidation, emphysema, interlobular septal thickening, bronchiectasis, fibrosis.

Disagreements between readers were resolved in a subsequent consensual reading session. The consensual analysis of the FD chest CT served as the reference standard to which the consensual analysis of ULD CT was compared.

### 2.7. Statistical Analysis

Quantitative variables were reported as average ± SD or median (1st quartile; 3rd quartile) according to their Gaussian distribution and assessed using the Shapiro–Wilk test. Categorical variables were reported as frequencies or percentages.

The inter-reader agreement for the subjective assessment of image quality was determined using Cohen’s kappa and quadratic weighting to account for the degree of disagreement. The confidence intervals for Cohen’s Kappa coefficients were calculated using the Bootstrap method. A kappa value of (0.0–0.20] was regarded as poor, (0.2–0.40] as fair, (0.41–0.60] as moderate, (0.61–0.80] as good and (0.81–1.00] as excellent.

Continuous variables were compared with a Student’s *t*-test when the parametric conditions were met, and otherwise with a Wilcoxon test.

The Pearson χ^2^ test or the Fisher exact test were used to compare the effectiveness of lesion detection (presence or absence) among the two groups, based on theoretical numbers.

Correlations between continuous variables were evaluated using the Spearman’s correlation coefficient. Comparison between the different correlation coefficients were made using the Hotelling Williams test.

Diagnostic performance of the ULD chest CT for the detection of 9 pulmonary lesions was compared to the FD chest CT: the sensitivity (Se), specificity (Sp), positive predictive value (PPV), negative predictive value (NPV), accuracy and error rate were calculated using a confidence interval of 95%.

A *p*-value smaller than 0.05 was considered statistically significant.

All statistical analyses were performed using R software, version 3.4.3. R Core Team (2015). (R: *A language and environment for statistical computing*. R Foundation for Statistical Computing).

## 3. Results

### 3.1. Population

A total of 170 patients were retrospectively included: 129 men (75.9%) and 41 women (24.1%). Age was 62.7 ± 11 years old (range 35–88).

The clinical indications were screening of asbestos-related pleuro-pulmonary diseases (*n* = 55), follow-up of pulmonary nodules (*n* = 39), pulmonary infections (*n* = 23), follow-up of lung cancer (*n* = 13), follow-up of interstitial pulmonary diseases (*n* = 10), follow-up of chronic obstructive lung diseases (*n* = 4) and other situations (*n* = 26).

The mean BMI was 26.6 ± 5.3 kg·m^−2^ (range 14.5–54.9), with a median BMI of 25.8 kg·m^−2^ (IQ 23.1–29.3). Of the patients, 45.9% (*n* = 78) had a BMI less than or equal to 25 kg·m^−2^; 33.5% (*n* = 57) had a BMI between 25 and 30 kg·m^−2^ and 20.6% (*n* = 35) had a BMI greater than or equal to 30 kg·m^−2^, of which eight patients (4.7% of the population) had a BMI greater than 35 kg·m^−2^.

The MTCD was 35.5 ± 3.4 cm (range 27–46).

### 3.2. Dosimetry

The DLP for FD acquisitions was 252.4 ± 143.2 mGy·cm (range 84.2–868.5), with a median of 204.4 mGy·cm (IQ 152.9–323.2). The average ED was estimated at 3.46 mSv.

The DLP for ULD acquisitions was 16.4 ± 1.8 mGy·cm (12.1–20.8), with a median of 16.3 mGy·cm (IQ 15.4–17.7). The average ED was estimated at 0.23 mSv, representing a 93% decrease compared to the FD acquisition.

### 3.3. Quantitative Image Quality

Image noise was 39.9 ± 11.3 for FD acquisitions and 53.3 ± 14.1 for ULD acquisitions, corresponding to a 33% increase.

**Figure 1:**
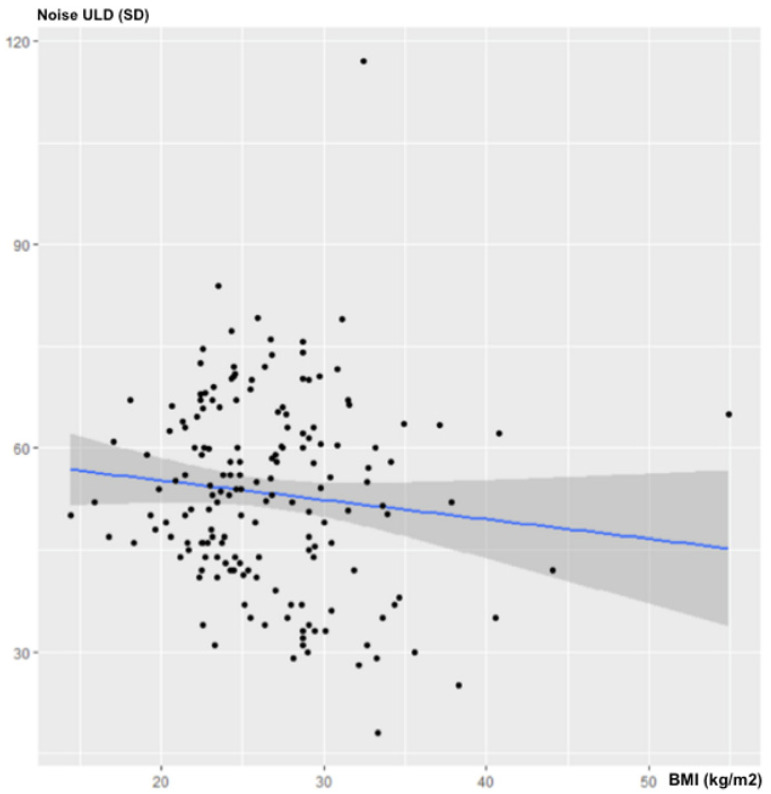
Influence of BMI on noise for ULD-CT.

There was no statistically significant correlation between BMI and noise in the ULD acquisitions: ρ = −0.12; 95% CI (−0.263–0.015); *p* = 0.11 (Figure 1).

**Figure 2:**
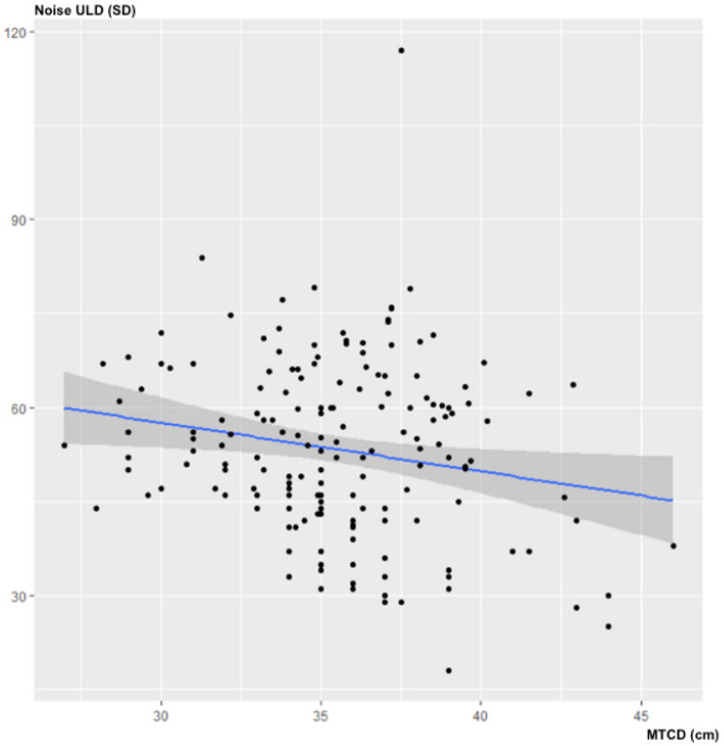
Influence of MTCD on image noise (in SD) for ULD-CT.

There was no statistically significant correlation between MTCD and noise in the ULD acquisitions: ρ = −0.14; 95% CI (−0.276–0.007); *p* = 0.07 (Figure 2).

### 3.4. Subjective Image Quality

**Figure 3:**
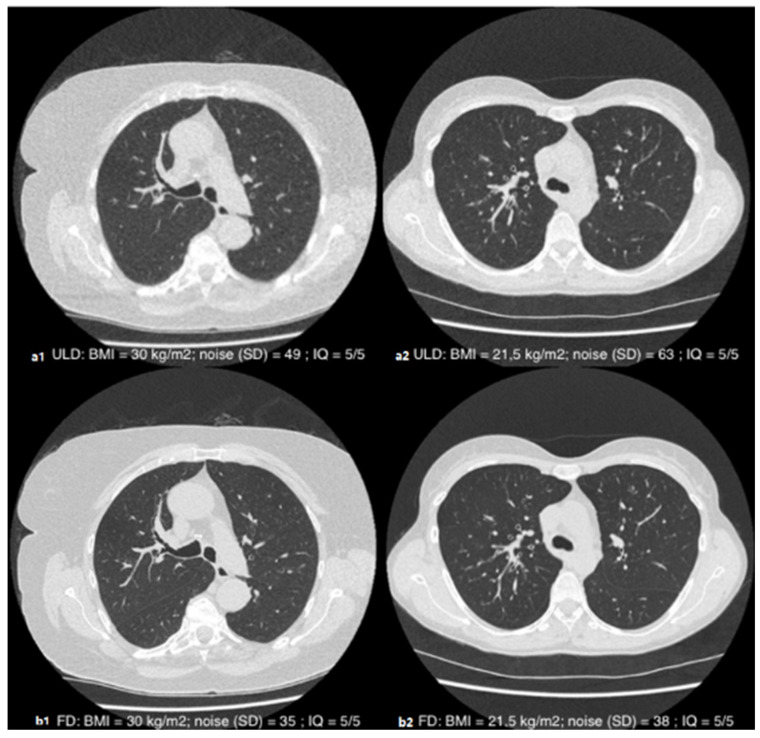
Axial slices in parenchymal window illustrating the subjective quality of the ULD (**a1**,**a2**) and FD (**b1**,**b2**) acquisitions in patients with a BMI of 30 kg·m^−2^ (1) and 21.5 kg·m^−2^ (2): diagnostic-quality images (QI = 5/5) despite the different morphotype.

The inter-reader agreement for the overall subjective image quality score was good, with a Cohen’s Kappa of 0.61 (95% CI [0.46–0.72]; *p* < 0.001). The average score assigned by the two readers for ULD image quality was ultimately 4.5 ± 0.6 with a median of 5 (4.5–5). The overall subjective image quality was almost always diagnostic (i.e., equal to or greater than three), except for two patients according to the junior reader (BMI at 44.1 and 37.8 kg·m^−2^) and one patient according to the senior reader (BMI at 44.1 kg·m^−2^). The distribution of the ratings is detailed in Table 3. An illustrative example is given in Figure 3.

**Figure 4:**
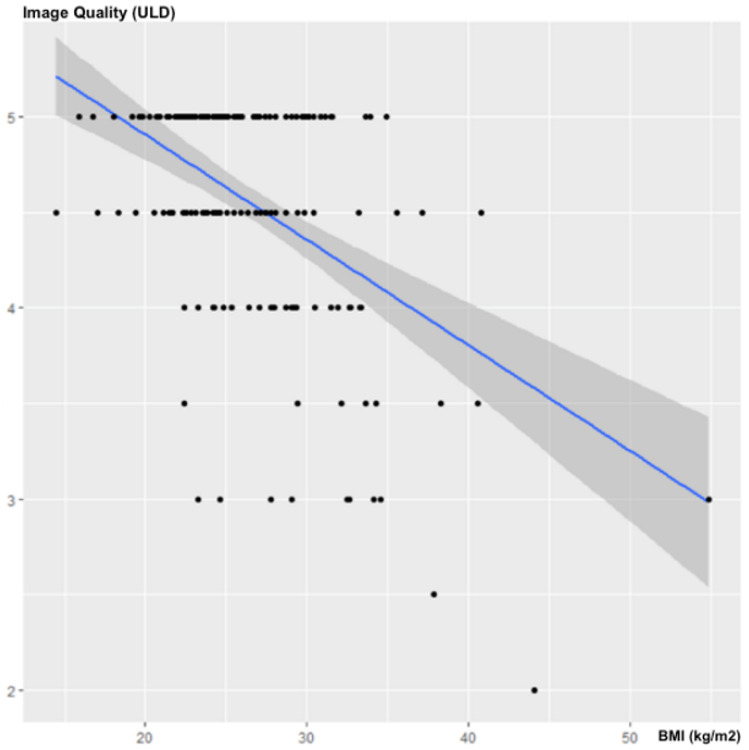
Influence of the BMI on subjective image quality.

The BMI was negatively and significantly correlated with the subjective image quality of the ULD acquisitions, with a correlation coefficient of ρ = −0.325; 95% CI (−0.462; −0.178); *p* < 0.001 (Figure 4).

**Figure 5:**
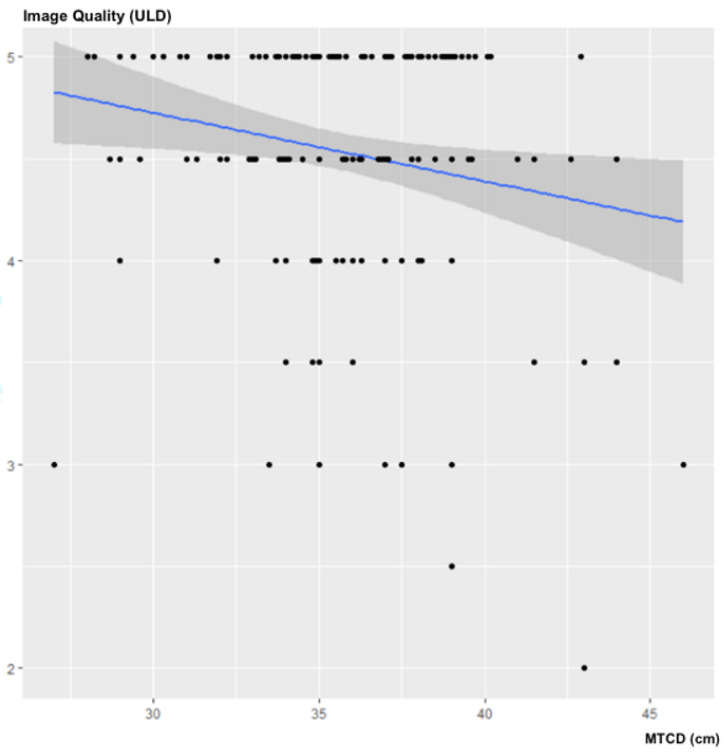
Influence of the MTCD on subjective image quality.

There was no statistically significant correlation between the MTCD and the subjective image quality of the ULD acquisitions: ρ = −0.0979; 95% CI (−0.253–0.055); *p* = 0.2041 (Figure 5).

### 3.5. Diagnostic Performance

At least one predefined parenchymal lesion was found in 114 (67%) out of the 170 examinations, with a total of 243 lesions on the reference FD acquisition. There was a disagreement in the reading of the reference CT scan in 44 patients: non-agreement rate = 25.9%; 95% CI, (19.5–33.2].

### 3.6. Diagnostic Performance per Patient

The overall diagnostic performance of the ULD chest CT (all lesions considered) were sensitivity, 77%; specificity, 99%; PPV, 94% and NPV, 65%.

The overall agreement rate for the interpretation of the ULD-CT scanner for all the lesions considered (disagreement = at least one difference out of nine lesions), was 68% (116/170) 95% CI [60.67–75.15].

There was no statistically significant correlation between BMI (*p* = 0.34), MTCD (*p* = 0.92) or gender (*p* = 0.53) and the per-patient diagnostic performance of the ULD-CT.

### 3.7. Diagnostic Performance per Lesion

The diagnostic performances per lesion are summarized in Table 4.

There was a statistically significant negative influence of the BMI on the diagnostic performance of the ULD CT for the detection of emphysema (*p* < 0.001). This negative correlation was not statistically found for the MTCD nor the gender.

There was no statistically significant correlation between BMI, MTCD or gender and the diagnostic performances for the eight other parenchymal lesions, provided that some lesions had a low incidence (ground glass nodules, masses, alveolar consolidation, septal thickening and fibrosis).

## 4. Discussion

Our study compiling 170 patients found an overall sensitivity of 77%, specificity of 99% and an agreement rate of 74.1% for a ULD chest CT when compared to the reference FD CT, using standard iterative reconstruction techniques. Despite the statistically significant negative correlation between image quality and BMI (ρ = −0.325; *p* < 0.001), we did not find a negative correlation between BMI and diagnostic performance (per patients or per lesions, except for emphysema). As a result, the deterioration in the image quality of a ULD CT associated with the increase in BMI does not translate into a significant decrease in diagnostic performance. Based on these findings, we can recommend the use of ULD CT, even when the BMI is greater than 30 kg·m^−2^, and likely up to 35 kg·m^2^ based on the BMI distribution in our population.

In prior publications, most studies did not include patients with a BMI greater than 30 kg·m^−2^.

In his study, Lee [11] compared the ULD CT (80 kV-30 mA fixed) to a low-dose (LD) CT in 81 patients, the average BMI of the population was 23.6 ± 3.8 kg·m^−2^ and only four patients had a BMI greater than 30 kg·m^−2^. They demonstrated a significant influence of BMI on the subjective image quality of the ULD CT (ρ = −0.480; *p* < 0.001), with the images being of diagnostic-quality in more than 95% of cases when the BMI was lower than 25, compared to only 70% when the BMI was greater than 25.

In the publication of a study conducted by Kim [13] using ULD CT (120 kV-15 mA fixed) in patients with neutropenic fever, all of the 207 patients included had a BMI lower than 30 kg·m^−2^ and only 14% of them had a BMI greater than 25 kg·m^−^^2^, among them four had a non-diagnostic image quality.

In Macri et al.’s study [16], using 100 kV and automated tube current modulation (60 mAs reference), a large majority of the patients included had a normal BMI (average BMI of 23.9 kg·m^−2^ for men and 23.1 kg·m^−2^ for women) and the image quality was excellent for 90% of the patients with a BMI up to 25, compared to only 76% of patients with a BMI greater than 25. It showed an excellent agreement between the ULD-CT results compared to LD-CT, except in two patients with a BMI at 26.8 and 27.4 kg·m^−2^.

Messerli et al. [12] studied the diagnostic performance of a ULD chest CT (100 kV, fixed tube current at 70 mAs, tin filtration) on the detection of pulmonary nodule compared to the reference FD CT. The population studied included 202 patients with a mean BMI of 26.2 ± 5.3 kg·m^−2^ (range 15.9–49), and the results showed a significantly decreased subjective image quality for the ULD CT in patients with a BMI greater than 30 (*p* < 0.001), in line with our findings. However, the number of patients with a BMI greater than 30 was not specified in the article. The authors found a statistically significant negative influence of the BMI on the sensitivity of the ULD-CT for the detection of pulmonary nodules: the sensitivity for the detection of all nodules was 92.6% in patients with a BMI of 25, compared to 84.9% in patients with a BMI of 35, which differs with our results.

To summarize, previous studies seldomly included obese patients (BMI > 30 kg·m^−2^) and always reported a significant deterioration in image quality for high BMI. Only nodules were analyzed, and conclusions were in favor of a negative correlation between diagnostic performance and BMI. Our work, which includes a significantly higher number of obese patients (35 patients) nuance these observations by confirming a negative correlation between subjective image quality and BMI, yet without negatively impacting the diagnostic performance for the detection of nodules and seven other parenchymal lesions.

There was no statistically significant correlation between the MTCD and the subjective image quality, confirming BMI as the only metric that can predict the image quality expected from a ULD CT examination. This is all the more useful since BMI is easily obtained before the acquisition. BMI and MTCD had an unexpected—even though not significant—tendency to lower the image noise. This positive influence could be explained by the high capacity of iterative reconstruction methods to significantly reduce noise. Similar results can be extracted from the literature [24,25], where images reconstructed with ASIR were associated with a greater noise reduction in high-weight patients.

The diagnostic performance of the ULD-CT was lower for emphysema and ground glass nodule compared to solid nodule and alveolar consolidation, which is in line with previous publications that have shown excellent diagnostic performance for spontaneously high contrast pulmonary lesions and lower performance for less attenuating lesions [11]. Unlike in the studies by Lee [11] and Macri [16], we chose to compare the diagnostic performance of the ULD CT to a reference “full dose” CT, which is the current Gold standard.

Several limitations of this work must be mentioned.

First, its retrospective nature—even if it is based on three prospective studies—and the relatively small number of severely obese patients (BMI > 35 kg·m^−2^)—even if this number is higher than in all the other publications on the subject.

Second, the population included in our study was mainly male, this is probably due to an inclusion bias for the 55 patients from the study that analyzed asbestos-related diseases and included only men. Nevertheless, the statistical analysis did not reveal any significant correlation by gender. Indeed, with breast attenuation being more significant in women, one could have imagined that at an equal BMI, the female sex could negatively influence the subjective image quality of the ULD CT.

Third, one third of the population was acquired in the prone position, which might have an impact on image quality.

Fourth, as a limitation inherent to all in vivo studies, reference was based only on the consensual reading of the FD CT by two readers, with a lack of any pathological confirmation. In the evaluation of subtle parenchymal lesions such as ground glass opacities, the analysis of multiple reviewers with different levels of expertise might have been more appropriate, as it could have led to a stronger consensus.

Finally, one should mention the monovendor nature of this study, carried out with a unique ULD acquisition protocol with a fixed tube current and a single iterative reconstruction algorithm. The prior publications listed used different iterative reconstruction software, and although our results are comparable to those studies, they cannot be strictly generalized to other constructors and other reconstruction techniques. Of note, the recently introduced Deep Learning Reconstruction might well further enhance the robustness of low dose acquisitions to high BMIs [26].

To conclude, our study suggests that despite a significant negative correlation of the BMI with the image quality of a ULD chest CT, its diagnostic performance is maintained. Consequently, the use of a ULD protocol when an unenhanced chest CT is needed could remain relevant in patients with a BMI greater than 30 kg·m^−2^.

## Figures and Tables

**Table 1:** Acquisition parameters of study protocols.

	ULD-CT	FD-CT (Reference)
**Voltage (kV)**	135	120
**Tube current (mA)**	10	80–700
**Tube current-time product (mAs)**	3	20–200
**Pitch**	0.813	0.813
**Rotation time (s)**	0.275	0.275
**Collimation**	0.5 × 80	0.5 × 80
**Reconstructed slice thickness (mm)**	1	1
**Reconstruction algorithm**	AIDR-3D	AIDR-3D

**Table 2:** Subjective image quality for ULD chest CT.

Subjective Image Quality		Ratings
**1**	Unacceptable image quality	*non-diagnostic examination*
**2**	Poor image quality	
**3**	Moderate image quality	*diagnostic examination*
**4**	Good image quality	
**5**	Excellent image quality	

**Table 3:** Distribution of ratings for subjective image quality.

Notes	R1	R2	Average
**1**	0 (0%)	0 (0%)	0
**2**	2 (1.2%)	1 (0.6%)	1.5
**3**	10 (5.9%)	19 (11.2%)	14.5
**4**	38 (22.3%)	51 (30%)	44.5
**5**	120 (70.6%)	99 (58.2%)	109.5
**Average ± SD**	4.62 ± 0.65	4.46 ± 0.71	4.5 ± 0.70

**Table 4:** Diagnostic performance of ULD-CT.

	Number of Lesions (*n*)	ULD-CT vs. FD-CT				Error Rate %
		Se (%)	Sp (%)	VPP (%)	VPN (%)	
**Abnormalities**	243	77 (188/243)	99 (1276/1287)	94 (188/199)	65 (860/1331)	25.9
**Solid nodule**	66	86 (57/66)	94 (98/104)	90 (57/63)	92 (98/107)	9
**95% CI**		[0.75; 0.93]	[0.88; 0.98]	[0.80; 0.96]	[0.84; 0.96]	
**Ground glass nodule**	8	62 (5/8)	99 (161/162)	83 (5/6)	98 (161/164)	2
**95% CI**		[0.24; 0.91]	[0.97; 0.99]	[0.36; 0.99]	[0.95; 0.99]	
**Mass > 3 cm**	3	100 (3/3)	100 (167/167)	100 (3/3)	100 (167/167)	0
**95% CI**		[0.29; 1]	[0.98; 1]	[0.29; 1]	0.98; 1]	
**Ground glass opacity**	30	70 (21/30)	99 (139/140)	95 (21/22)	94 (139/148)	6
**95% CI**		[0.51; 0.85]	[0.96; 0.99]	[0.77; 0.99]	[0.89; 0.97]	
**Alveolar consolidation**	24	83 (20/24)	99 (144/146)	91 (20/22)	97 (144/148)	4
**95% CI**		[0.63; 0.95]	[0.95; 0.99]	[0.71; 0.99]	0.93; 0.99]	
**Emphysema**	58	81 (47/58)	99 (111/112)	98 (47/48)	91 (111/122)	7
**95% CI**		[0.69; 0.90]	[0.95; 0.99]	[0.89; 0.99]	[0.84; 0.95]	
**Interstitial septal thickening**	11	45 (5/11)	100 (159/159)	100 (5/5)	96 (159/165)	4
**95% CI**		[0.17; 0.77]	[0.98; 1]	[0.48; 1]	[0.92; 0.99]	
**Bronchiectasis**	31	71 (22/31)	100 (139/139)	100 (22/22)	94 (139/148)	5
**95% CI**		[0.52; 0.86]	[0.97; 1]	[0.85; 1]	[0.89; 0.97]	
**Fibrosis**	12	67 (8/12)	100 (158/158)	100 (8/8)	98 (158/162)	2
**95% CI**		[0.35; 0.90]	[0.98; 1]	[0.63; 1]	[0.94; 0.99]	

## Abbreviations

BMI	Body Mass Index
CT	Computed Tomography
DLP	Dose length Product
ED	Effective Dose
FD	Full Dose
MTCD	Maximum Transverse Chest Diameter
ROI	Region Of Interest
SD	Standard Deviation
ULD	Ultra-Low Dose
